# Unemployment benefits, entrepreneurship policies, and new business creation

**DOI:** 10.1007/s11187-023-00735-9

**Published:** 2023-02-28

**Authors:** Irina Bilan, Constantin-Marius Apostoaie

**Affiliations:** Department of Finance, Money and Public Administration, Faculty of Economics and Business Administration, Alexandru Ioan Cuza University of Iaşi, Blvd. Carol I No. 11, 700506 Iaşi, Romania

**Keywords:** Unemployment benefit replacement rate, Nascent entrepreneurship, Opportunity and necessity entrepreneurship, Entrepreneurship programmes and support, European Union countries, L26, M13, H53

## Abstract

Besides their common link with unemployment, unemployment benefits are also relevant to the decision to become an entrepreneur. We thoroughly explored this relationship for a panel of 23 EU countries over the period 2001–2019. Our results demonstrate that generous unemployment compensations hinder entrepreneurial initiative, and those opportunity entrepreneurs, who are more likely to create new jobs and innovation, are affected more. Contrary to common belief, we find an unequal pattern of effects, with higher benefits being more detrimental at the beginning of the unemployment spell. A favourable policy framework results in being relevant for entrepreneurial endeavours on its own; in addition, high-quality policies and programmes for entrepreneurs are found to temper the negative effects of large unemployment benefits on new business creation during long unemployment spells. Our results support the call for properly designed unemployment benefit systems (as both level and time pattern) that ensure an optimum balance between adequate income replacement and poverty prevention, on the one hand, and limited side effects on new venture creation, on the other hand.

**Supplementary Information:**

The online version contains supplementary material available at 10.1007/s11187-023-00735-9.

## Abstract (plain language summary)

Generous unemployment payments can either “make or break you”! In simple words, while social benefits may contribute to income support and poverty prevention among the unemployed, these can also harm the economy by breaking one of its most important engines: entrepreneurship. By focusing on 23 EU countries, the paper highlights a negative impact of unemployment compensations on overall but also opportunity entrepreneurs, while the effects on necessity entrepreneurs are inconclusive. New business creation is inhibited when unemployment benefit systems offer generous compensations, especially at the beginning of the unemployment spell. At long unemployment durations, high-quality policies and programmes for entrepreneurs efficiently act towards diminishing such side effects. Our findings suggest that, when choosing the design features of social security systems, policymakers should definitely consider their adverse impact on entrepreneurship. Even when unemployment benefits are large, their side effects could be limited by compensatory measures, such as stricter job-search requirements or allowing unemployed individuals to keep receiving compensation while in the process of creating a new business.

**Supplementary Information:**

The online version contains supplementary material available at 10.1007/s11187-023-00735-9.

## Introduction

Entrepreneurship is widely recognised as a major driver of economic growth and development in a country, which can impact the economy and society in numerous and complex ways, providing new job opportunities and reducing unemployment, promoting innovation, triggering social change, and improving the well-being of people. The positive role of entrepreneurship motivated researchers to put great effort into investigating the factors that spur it or act as a barrier or deterrent for the creation of new businesses, and policymakers to continuously redesign and adjust public policies to support it. In particular, an examination of the strategies and measures that are taken worldwide to overcome the effects of the recent pandemic crisis reveals that policymakers and other interested parties at local, national, European, and international levels are looking towards entrepreneurship as a panacea for the problem; as such, important amounts of resources are geared towards either the creation of new businesses or the development of the existing ones that could spur positive and long-lasting outcomes.^1^

In most of the European Union (EU) Member States, as in other developed and emerging economies, there is a long tradition of generous welfare systems. Unemployment benefits are a key feature of these systems, meant to provide individuals with protection against the risk of income loss and poverty because of unemployment. In the EU, the design characteristics of unemployment insurance schemes are certainly very diverse and subject to change over time.^2^ Since, over the past decades, social security systems have continuously increased in complexity, it became obvious that they may impact the economy and society in new and sometimes unexpected ways, and impressive efforts have been made so far to comprehend and assess their effects. In particular, it has been ascertained that specific features of the unemployment benefits systems strongly affect unemployed individuals’ behaviour and employability, and these outcomes call for thorough investigations so as to substantiate new policies limiting undesired results.

Our study seeks to investigate the relationship between the generosity of unemployment benefit systems and new business creation in EU countries. We conduct our analysis over a period of 19 years (2001–2019), in which unemployment protection schemes were subject to various changes in response to the global economic and financial crisis of 2007–2009, the sovereign debt crisis, and as part of the post-crisis fiscal consolidation strategies in Europe.

With rising unemployment rates and increased support for unemployed workers during the crisis of 2007–2009 and the recent COVID-19 pandemic, research on the effects of unemployment benefit systems resumed momentum. However, the bulk of the literature seems to focus on the interplay between unemployment benefits and unemployment (among others: Chetty, 2008; Ekkehard, 2015; Farber et al., 2015; Rebollo-Sanz & Rodríguez-Planas, 2020; Petrosky-Nadeau & Valletta, 2021), and mostly provides evidence that extensive unemployment entitlements, as level or duration, and weak job-search and availability-to-work requirements to keep receiving benefits inhibit job search incentives and reduce unemployment exit rates. That being said, much less attention has been paid to the effects of unemployment benefit systems on entrepreneurial activity in a country, although a few papers directly or indirectly explore this strand of research (Parker & Robson, 2004; Kanniainen & Vesala, 2005; Hessels et al., 2007; Koellinger & Minniti, 2009; Robson, 2010; Røed & Skogstrøm, 2014; Xu, 2022). As most empirical studies evidence a negative relationship between generous unemployment benefits and entrepreneurial indicators, this might have led many to believe that the same findings in the unemployment literature apply to entrepreneurship and there is no need for further, in-depth investigation. It is within this niche of the literature that our paper brings most of its contributions (with more details below).

Current entrepreneurship literature does not seem to provide a comprehensive theoretical framework for the relationship between unemployment benefits and new business creation that explores the different channels through which the two may be connected. It usually just reiterates the main labour market argument related to the opportunity costs of higher unemployment benefits for leaving unemployment (both for a paid job and taking the risks associated with creating a new business) or for choosing self-employment to wage employment (Wennekers et al., 2005; Koellinger & Minniti, 2009; Robson, 2010; Røed & Skogstrøm, 2014). Hence, we focus our attention in the following section on providing a comprehensive depiction of the various channels through which the design features of unemployment benefit systems may affect a person’s decision to become an entrepreneur and new firm creation in a country.

Entrepreneurs choose this career path for different reasons; some are motivated by good business opportunities to start a new venture, and others are “pushed” into entrepreneurship by financial constraints, hence opportunity-driven vs. necessity-driven entrepreneurs (Wennekers et al., 2005; van der Zwan et al., 2016; Audretsch et al., 2022). Unfortunately, there is only scarce and inconsistent evidence with regard to the effects of social security systems across the two types of entrepreneurs (Hessels et al., 2008b; Koellinger & Minniti, 2009; Audretsch et al., 2022). The finding of a positive relationship between social security variables and necessity entrepreneurs in the studies of Hessels et al. (2008b) and Audretsch et al. (2022) does not support the core theoretical argument that more generous social benefit systems discourage individuals from becoming entrepreneurs out of necessity by providing them with alternative economic means. Hessels et al. (2008b) explain that higher social security contributions result from many beneficiaries and scarce job opportunities (therefore, high unemployment), an explanation that is not consistent with the usual practice of governments not increasing social security contributions under unfavourable labour market conditions. Our study sheds some light on these dilemmas and brings its contribution to the literature by revealing to what extent generous unemployment benefits affect the creation of new businesses in different ways, depending on what motivates an individual to start up a new venture.

The design of unemployment benefit systems differs between countries, but it is common for unemployment compensations to decrease with the time spent out of the labour market. In the unemployment literature, there is a wide consensus that generous unemployment benefits at longer unemployment spells are more harmful to incentives than in the early stages of unemployment (Cahuc & Lehmann, 2000; Tatsiramos & van Ours, 2014); however, some recent studies clearly contradict this evidence (Kolsrud et al., 2018; D’Ambrosio & Scrutinio, 2022). In the entrepreneurship literature, the time pattern of unemployment benefits’ impact on entrepreneurial endeavours received (to our best knowledge) no attention so far. Our study brings its contribution by looking at the nexus between unemployment benefits and entrepreneurial initiatives at different unemployment spells. This endeavour is motivated by the practical value of designing policy proposals aimed at diminishing the adverse effects of existent welfare systems.

Social security systems and unemployment benefits, in particular, are part of the wider institutional environment in a country, where different institutional features interact. The role of government policies in promoting entrepreneurship is well ascertained, and many studies investigate how tax policies, subsidies, or active labour market policies shape the decision to become an entrepreneur and the survival and performance of new businesses (e.g. Kumar, 2012; Roman et al., 2013; Acs et al., 2016; Schmieder & von Wachter, 2016; Barbosa et al. 2017). However, evidence on the interaction between unemployment benefit systems and the wider entrepreneurship policy framework in which entrepreneurial ideas emerge and turn into action is scarce, focusing almost exclusively on active labour market policies (Laffineur et al., 2017; Schmieder & Trenkle, 2020). As suggested by Schmieder and Trenkle (2020), we believe that such interactions should be furtherly investigated, as they may lead to biased estimates of unemployment benefits’ effects. Hence, our paper brings its contribution by revealing whether the quality of entrepreneurial framework conditions, in particular of government policies and programmes for entrepreneurs, moderates the impact of unemployment compensations on the dynamics of new business creation.

To sum up, our study adds to the literature on entrepreneurship determinants and on the economic effects of social security systems in several major ways. First, from a theoretical perspective, it draws a comprehensive and clear picture of the various ways and channels through which the design features of unemployment benefit systems may affect a person’s decision to become an entrepreneur and new firm creation in a country. Second, it empirically investigates not only the overall effects of generous unemployment benefits (measured by the net replacement rates between unemployment payments and wage earnings in employment) on entrepreneurial activity in a country but also how these effects differ along the unemployment spell. As such, it contributes to the growing literature strand on the dynamic features of social security systems by providing compelling evidence that these features matter not only for employment incentives but also for entrepreneurial initiatives. Third, it explores the differences in the impact of generous unemployment benefit systems on necessity and opportunity entrepreneurs, for which only scarce and inconsistent evidence exists. As it is widely acknowledged that opportunity entrepreneurs are more innovative, create more jobs, and contribute more to economic growth (Rodrigues & Teixeira, 2020), additional findings on this distinction could contribute to designing better policies that spur this type of entrepreneurship in particular. Finally, it evaluates the moderating role of the wider entrepreneurship policy framework on the relationship between unemployment benefits and new business creation, an issue which received only a little attention so far.

The remainder of this paper is structured as follows: Section 2 explores the main theories and arguments that explain the effects of unemployment compensations on new venture creation and how they may be influenced by the wider entrepreneurship policy framework and draws the research hypotheses; Section 3 presents the data, models, and variables that we used in our empirical investigation; Section 4 presents the results and some robustness checks to test their sensitivity to model specification changes and alternative measures of unemployment benefit generosity; the final section concludes and provides some policy recommendations.

## Theoretical underpinnings and hypotheses development

### Unemployment benefits and the creation of new businesses

While the effects of old age, sickness, disability, or other social security arrangements on the emergence of entrepreneurial activities in a country are less obvious, the same does not apply to unemployment benefits. Designed as safety nets against the risk of losing one’s job, unemployment benefit schemes are inherently connected with the functioning of the labour market and may influence a person’s occupational choice and willingness to take the risks associated with becoming an entrepreneur.

#### Unemployment insurance as a safety net for business failure

Starting a business can be a very risky endeavour, and not all individuals are willing to assume high risks. Therefore, entrepreneurship may not be a feasible option for all people. Designed to offer protection against the risk of income loss, social security systems may increase individuals’ appetite to engage in riskier activities (Robson, 2010; Audretsch et al., 2022). Unemployment insurance schemes may act as a safety net in case of business failure and encourage individuals to explore the riskier entrepreneurship path (Wennekers et al., 2005; Hessels et al., 2007; Robson, 2010; Rapp et al., 2018). More generous unemployment compensations could, therefore, be associated with a higher prevalence of entrepreneurship.

Not only risk-averse but also risk-tolerant persons could see entrepreneurship as a more feasible option, under the condition that unemployment insurance systems provide coverage to self-employed workers and salaried employees alike (Rapp et al., 2018). However, in many countries, this is an ongoing issue, as self-employed persons are only partially covered or not at all (International Labour Office, 2019).^3^

#### Unemployment benefits and occupational choice

Three main situations can characterize an individual’s occupational status at one moment in time: he could work as a salaried employee, be self-employed, or be unemployed. Wage-earning persons could also choose to start a new business; nonetheless, theory shows that the pool of unemployed individuals is, in its turn, a major source of entrepreneurial initiatives (Kumar, 2012; Røed & Skogstrøm, 2014), and several studies even suggest that people without a job have a higher probability of becoming entrepreneurs than employed persons (Kuhn & Schuetze, 2001; Berglann et al., 2011). Losing their job may push some individuals to become entrepreneurs out of necessity because there are no good enough job opportunities and they have no other means of earning income (Blanchflower, 2004).

Although there are several alternative exit routes from unemployment (among which early retirement, ill-health retirement, and maternity/paternity leave), two major options are available to the regular unemployed individual: to find a new temporary or permanent job in existing firms or open a new business venture. The existing system of unemployment compensation in a country may affect a person’s willingness to leave unemployment for both salaried employment and self-employment. In terms of opportunity costs, more generous unemployment benefits (in particular, higher unemployment compensations relative to wages) mean higher costs for leaving unemployment, and individuals are encouraged to lessen their efforts to find a paying job or create a new venture that they will own or co-own (Robson, 2010; Laffineur et al., 2017). A moral hazard effect could occur (Chetty, 2008) as unemployed persons choose to stay unemployed for longer or just act in the proximity of the expiration date of unemployment compensations. Therefore, the higher the unemployment benefits are, the less entrepreneurial initiative is expected to exist in a country.

The design of the unemployment insurance system matters in this equation. Disincentives are stronger when benefits are lost as soon as the unemployed individual gives up looking for a job and starts a new venture, or when business income is deducted from unemployment benefits, and milder when the benefits are still available for the individual while in the process of creating his own business (Hombert et al., 2020; Xu, 2022). Moreover, social security coverage could be equally relevant to occupational choice. Less generous unemployment benefits or no coverage for the self-employed increase the opportunity costs of choosing to become an entrepreneur and inhibit entrepreneurial endeavours (Wennekers et al., 2005; Hessels et al., 2007). This holds true for both unemployed and wage-earning individuals who are thinking about exploring an entrepreneurial career. Generous unemployment benefits, in particular a high unemployment replacement rate, may also discourage salaried workers from leaving paid employment to work on their own because of fear of benefit loss (Parker & Robson, 2004; Laffineur et al., 2017).

#### Unemployment benefits, efficiency wage, and business costs

For employed individuals, unemployment benefits are an outside option that shapes their work behaviour, reducing work incentives (Beladi & Kar, 2014; Schmieder & Wachter, 2016). In the framework of the efficiency wage model of Shapiro and Stiglitz (1984), unemployment compensation could be considered a short-term alternative option for people who get fired for shirking at work. In the presence of generous unemployment benefits, workers are induced to shirk more, and companies incur larger monitoring costs to prevent shirking. In the end, firms may also have to offer efficiency wages to reduce losses due to shirking (Kumar, 2012; Beladi & Kar, 2014), and these higher costs can hurt entrepreneurs. For the potential new incomers, faced with high financial constraints, additional costs might prove to be prohibiting and discourage them from engaging in any kind of entrepreneurial activity at all.

On the same path of reasoning, when unemployment benefit replacement ratios are high, this raises the reservation wage, and some individuals might choose to voluntarily become unemployed (Beladi & Kar, 2014; Laffineur et al., 2017). Firms may have to offer higher than optimal wages, and entrepreneurial activities are inhibited (Beladi & Kar, 2014). Moreover, generous unemployment compensations come hand in hand with high taxes and social contributions, depending on the way benefits are funded in a country, and this additional tax burden may inhibit new firm creation even more (Hessels et al. 2008a, b).

The above theoretical arguments may unequally apply to necessity and opportunity entrepreneurs or at different timings along the unemployment spell, issues that are further explored in Sections 2.2 and 2.3. Based on them, we hypothesise that:

- H1: A more generous unemployment benefit system in a country discourages individuals from starting up new businesses.

### Time profile of unemployment benefits and new business creation

The effects of unemployment compensations at different timings during the unemployment spell on entrepreneurial endeavours received, to our best knowledge, no attention in the literature, but some relevant arguments can be drawn from the extensive unemployment research, as for an unemployment benefit recipient both self-employment and paid employment are possible alternatives to unemployment.

In this area, the conventional view is that large unemployment benefits at longer unemployment spells are more harmful to incentives than in the early stages of unemployment (Cahuc & Lehmann, 2000; Tatsiramos & van Ours, 2014). This view is reflected in the architecture of unemployment benefit systems in many countries, being quite common for the generosity of compensation schemes to decrease with the time spent in unemployment. Unemployment insurance benefits, depending on previous contributions, are relatively high but available for only a limited period, and in some cases, start to diminish for longer unemployment durations. Social assistance benefits for the unemployed, which offer only minimal compensation for income loss, are sometimes available when people are not (or no longer) eligible for unemployment insurance payments. Overall, this leads to a declining structure of unemployment benefits that is said to create more incentive to search for a job (Cahuc & Lehmann, 2000) or pursue entrepreneurship.

Opposing the conventional view, an emerging and still scarce strand of literature on the optimal timing of unemployment benefits (Kolsrud et al., 2018; D’Ambrosio & Scrutinio, 2022) provides evidence for a lower moral hazard cost of changes in benefit generosity and smaller negative impact of unemployment compensations at longer unemployment durations and supports for a non-declining benefit profile.

One possible argument shows that, along the unemployment spell, the behaviour and expectations of unemployed individuals are subject to change, further affecting their search for job endeavours and entrepreneurial actions. Nikiforou et al. (2019) point out that the level of need increases when a person stays unemployed for longer, as individuals may feel a higher psychological need for personal fulfilment and higher pressure of shrinking skills and social capital depreciation (Storey, 1991; Nichols et al., 2013). This decay in human and social capital at long-term unemployment durations may result from both changes in the labour market, which moves farther away from the skills and qualifications at previous work, and changes in the workers’ behaviour, who look farther afield for a new job (Nichols et al., 2013).

The increased pressure to get out of unemployment could make unemployed individuals lower their expectations, accept jobs beneath their qualifications and smaller wages, or reconsider the entrepreneurial path. Under such pressure, at longer unemployment spells, the deterring effect of generous unemployment benefits on start-up incentives might be lower. Entrepreneurial know-how and programmes for entrepreneurship training that smooth the transition from unemployment to self-employment play an important role at this point, as is generally believed that formerly unemployed individuals lack the basic qualifications to become entrepreneurs (Caliendo & Kritikos, 2010).

From another perspective, unemployment benefit schemes are meant to temporarily create access to alternative sources of income when labour income is no longer available, but unemployed individuals might also live on other means like savings and transfers, and the availability of these means may alter the effects of unemployment benefits over time. At short-term unemployment durations, such means could be more important and diminish the incentive to search for a new job or become self-employed out of necessity when unemployment compensations are low. This is especially relevant in high-income countries with high levels of household savings and individual wealth. In less developed countries, with cultural systems oriented towards high family and/or community support, solidarity at this level could play a similar role in the short run (Margolis, 2014). Nevertheless, generous benefits in the very early stages of unemployment may also serve as an additional source of funding for new entrepreneurs who see good opportunities to start a business.

However, at longer unemployment durations, as savings run out and little alternative means are available, individuals may be more constrained to look for a job or start a business out of necessity. The pressure of no resources or financial capital depreciation increases (Storey, 1991). In simple terms, at longer unemployment spells, unemployed individuals might depend more on social transfers as means of economic survival. The sensitivity of entrepreneurial and job search behaviour to the level of unemployment compensations increases, and higher benefit payments are more likely to reduce the incentive to create a new business venture, especially out of necessity.

Different driving forces may play a role in the interplay between unemployment benefits and entrepreneurial initiative at different unemployment spells, leading to a possible unequal pattern of effects over time, which received little attention in the entrepreneurship literature but could be very relevant from a policy-making perspective. Based on the conventional view on unemployment benefits’ effects over time, we hypothesize that:

- H2: There is an unequal time pattern of unemployment benefits’ effects on new business creation, with stronger effects of generous compensations at later stages of unemployment.

### Unemployment benefits and opportunity and necessity entrepreneurship

An individual may choose an entrepreneurial career for several reasons, and previous studies suggest that the explanatory factors of entrepreneurship may differ depending on the entrepreneur’s motivation, aspirations, and choice of the sector (Hessels et al., 2008b; Thai & Turkina, 2014; van der Zwan et al., 2016; Audretsch et al., 2022). One taxonomy, in particular, is getting a substantial amount of attention in the literature that distinguishes between opportunity and necessity entrepreneurs (Wennekers et al., 2005; Koellinger & Minniti, 2009; Margolis, 2014; van der Zwan et al., 2016; Fairlie & Fossen, 2020; Rodrigues & Teixeira, 2020; Audretsch et al., 2022). The basic distinction is that the former are motivated to start a new venture in order to exploit potential business opportunities, while the latter decide to become entrepreneurs because they have no good-enough alternatives in the labour market and no other means of earning income (Wennekers et al., 2005; Fairlie & Fossen, 2020). Opportunity entrepreneurs decide to start a business as a choice among several possible career options, but for necessity entrepreneurs, this is a last-resort option, other alternatives being non-existent or unsatisfactory (Wennekers et al., 2005; Acs et al., 2008; Audretsch et al., 2022). Because of what triggers their entrepreneurial initiative, necessity entrepreneurs tend to be much more sensitive to financial incentives (Røed & Skogstrøm, 2014).

The different motivations for starting a business are closely related to the previous occupational status of entrepreneurs (Hessels et al., 2008b; Belda & Cabrer-Borrás, 2018; Fairlie & Fossen, 2020), further determining the way and channels through which generous unemployment benefits impact entrepreneurial endeavours. Hessels et al. (2008b) link the necessity motive to (a threat of) unemployment. In their quest for an operational definition of opportunity versus necessity entrepreneurship, Fairlie and Fossen (2020) propose, in agreement with the standard economic model of entrepreneurship, to consider necessity entrepreneurs as individuals who are unemployed before setting up a new business, and opportunity entrepreneurs those who are wage-earning persons, enrolled in school or college, or not actively seeking for a job. Belda and Cabrer-Borrás (2018) also consider opportunity entrepreneurs to be those who voluntarily leave their job to create a new business, and necessity entrepreneurs those who start up a business after losing their job or who become self-employed after being unemployed for more than 6 months.^4^

Therefore, necessity entrepreneurs usually make a transition from unemployment to entrepreneurship, and the level of social security benefits that they are entitled to while being unemployed directly influences their occupational choice. When generous unemployment benefits provide them with enough means to survive, the “push” to pursue the entrepreneurial path in the absence of alternative job options may be weaker, and recipients may choose to stay unemployed for longer and keep looking for a job as their preferred option. This is especially important in countries with strong social security systems that offer good conditions for pursuing employment (Laffineur et al., 2017). Disincentives may, however, become weaker close to the expiration date of unemployment compensations (Chetty, 2008), when no further social benefits (e.g. social assistance) are available at a reasonable level and the unemployed feel more pressure from imminent loss of economic means and are forced to take action. Røed and Skogstrøm (2014) clearly demonstrate that there is a significant increase in entrepreneurship hazard close to the exhaustion of unemployment insurance benefits.

The same reasoning does not generally apply to opportunity entrepreneurs, who more often start a business before leaving a salaried job or make a direct transition from employment to business ownership (Belda & Cabrer-Borrás, 2018). The voluntary resignation from their previous job to pursue entrepreneurship or failure to actively look for a job while in the process of creating a new business should not entitle them to unemployment benefits. However, in this case, generous social security benefits may still act as opportunity costs for leaving paid employment (Wennekers et al., 2005; Hessels et al., 2007) and discourage potential entrepreneurs who seize good business opportunities to take action, especially when there is little or no social security coverage for the self-employed.

From another perspective, previous evidence suggests that there are substantial differences in the type of business venture that opportunity and necessity entrepreneurs create and the aspirations they have for the firm (Williams, 2007; Hessels et al., 2008b; Fairlie & Fossen, 2020), and these differences become more important at longer unemployment spells of necessity entrepreneurs (Nikiforou et al., 2019). New opportunity entrepreneurs are more likely to create incorporated businesses (Fairlie & Fossen, 2020), which operate in the formal sector where taxes and social contributions have to be paid. Higher social contributions and taxes raised to provide extensive social security benefits may be seen by opportunity entrepreneurs as cutting into their future profits and reducing their expected returns. These create incentives for opportunity entrepreneurs to spend less and delay investment and may even discourage individuals who see good business opportunities from entering the market (Audretsch et al., 2022). Hessels et al. (2008b) find employer compulsory social contribution rates to negatively impact the prevalence of innovative entrepreneurship, which closely relates to opportunity entrepreneurship. Forced out of the labour market, necessity entrepreneurs are more likely to create unincorporated businesses or be engaged in entrepreneurship in the informal sector (Williams, 2007), where tax policy has a lesser effect on entrepreneurial decisions and there is less pressure of losing unemployment benefits while in the process of creating a new business. Nonetheless, unincorporated businesses usually have smaller profits compared to incorporated businesses, and, therefore, the decision to create such a business is more strongly affected by generous unemployment benefits when new business income is deducted from these benefits (Xu, 2022).

New opportunity entrepreneurs are more growth-oriented and more likely to create employer businesses (Fairlie & Fossen, 2020). When hiring salaried workers to perform their daily business activities, their expected profits strongly depend on the dynamics of wages. Higher than optimal reservation wages that may have to be paid when generous unemployment benefits are available to workers as an alternative short-term option raise the wage bill and diminish expected returns from entrepreneurship, inducing marginal opportunity entrepreneurs to give up starting a business and choose employment instead. Depending on their business for daily economic survival, necessity entrepreneurs usually have lower expectations for innovation and growth in terms of jobs created, as they are aware that such ambitions would be difficult for them to accomplish (Hessels et al., 2008b). They are more often solo workers (Roman et al., 2013; Fairlie & Fossen, 2020; Dvouletý, 2022) and do not have the alternative option of a paying job; therefore, the same reasoning does not apply.

Based on the above theoretical arguments, both necessity and opportunity entrepreneurship are expected to be affected by generous unemployment benefit systems, although for different reasons and through different channels, and we hypothesize that:

- H3(a) A more generous unemployment benefit system in a country discourages opportunity entrepreneurs from starting up new businesses;
- H3(b) A more generous unemployment benefit system in a country discourages necessity entrepreneurs from creating new businesses.

### The role of government policies and programmes for entrepreneurs

In their quest for long-term development and prosperity, governments are more than motivated to design policies, programmes, and regulations and direct their resources towards actions targeting entrepreneurial endeavours. This is a clear path to job creation and economic growth (Roman et al., 2013; Chowdhury et al., 2019; Rodrigues & Teixeira, 2020), but also a means to better manage social issues like exclusion, inequality, and poverty (Parker, 2009). Government policies play a major role in shaping the driving forces that may trigger or inhibit entrepreneurial activity in a country. They act in various and complex ways, and extensive literature develops around the effects of regulations and direct actions through taxes, subsidies, and labour market programmes (among others: Storey, 2003; Cullen & Gordon, 2007; Caliendo & Kritikos, 2010; Røed & Skogstrøm, 2014; Arin et al., 2015; Barbosa et al., 2017; Chowdhury et al., 2019; Audretsch et al., 2022). The entrepreneurs’ perception of such institutional environment features counts just as much as the objective environmental conditions for entrepreneurial decisions (Edelman & Yli-Renko, 2010).

The government may contribute to a better entrepreneurial environment by funding public activities that increase the quality of human capital, such as schools, informal educational programmes, nonformal training programmes, and healthcare programmes. In addition, the government can finance physical infrastructure (such as roads, transport, access to utilities and internet, provides free working spaces) as well as institutional infrastructure (chambers of commerce, land and property rights, and intellectual property protection) with the clear aim to enhance business creation. Overall, public resources can be oriented towards enhancing the inputs of an entrepreneur, generating a ‘pull’ effect. Moreover, governments can channel their finances towards offering social security and welfare programmes that mitigate risks and lower the opportunity costs of going into business instead of wage employment (see Audretsch et al., 2022).

On the same note, taxes are shaping the entrepreneurial environment worldwide (Cullen & Gordon, 2007; Chowdhury et al., 2019; Audretsch et al., 2022). In simple terms, paying taxes always discourages profit-seeking entrepreneurs. However, one should not oversimplify their effects, as the literature has identified complex channels through which tax policy and entrepreneurship interact (Cullen & Gordon, 2007, speak, for example, about income shifting, risk subsidy, and risk-sharing). Under generous government support for entrepreneurs (providing significant public goods and services), a burdensome tax policy may not discourage individuals from creating new businesses. Audretsch et al. (2022) demonstrate, in this respect, that a mix of the large government sector and high taxes may, in fact, create conducive conditions for entrepreneurs, as they have greater access to resources. In addition to tax regulations, other specific regulatory policies, such as labour regulations, contract enforcement, bankruptcy laws and licensing, and environmental policies, may shape the entrepreneurial initiative (Braunerhjelm et al., 2015; Chowdhury et al., 2019).

Directly targeting entrepreneurs, a government can develop particular programmes subsidising overall or specific businesses (e.g. high-quality and society-oriented start-ups). Storey (2003) identifies several particular areas where such programmes are relevant for entrepreneurs: finance, markets/demand, administrative burdens, premises, new technology, and skilled labour. In practice, these programmes are heterogeneous among countries; nonetheless, they are more fine-tuned and better targeted and may contribute not only to increasing the quantity but also the quality of entrepreneurship (Chowdhury et al., 2019).

A particularly important set of programmes, with origins in the wider active labour market policies (ALMPs), are start-up programmes for the unemployed. Although directly targeted at reducing unemployment, they may have a major contribution to spurring entrepreneurial initiatives among the unemployed (Caliendo & Kritikos, 2010; Røed & Skogstrøm, 2014). Among others, they can improve access to financial capital, make people less dependent on loans, or improve individuals’ entrepreneurial skills. However, evidence on the effectiveness of such programmes is mixed, depending on the type of entrepreneurship and quality of other labour market institutions (Laffineur et al., 2017), the economic situation, or the stringency of labour laws (Roman et al., 2013).

ALMPs dedicated to promoting entrepreneurship are evidenced to be more efficient when they jointly act with adequate social security systems that do not create disincentives for unemployed individuals to take risks and become entrepreneurs. In particular, high rigidities are induced by inadequate unemployment insurance systems, which offer overgenerous benefits (Laffineur et al., 2017; Schmieder & Trenkle, 2020) or lack adequate job search requirements and sanctions (Røed & Skogstrøm, 2014).

Overall, wide policy support for entrepreneurs and specific entrepreneurship programmes can help people overcome the different barriers and challenges of creating a new business and spur entrepreneurship. Under such circumstances, the opportunity costs and other business costs related to generous unemployment benefits could seem less relevant to individuals who are thinking of starting up a new venture, and they will be less discouraged in their entrepreneurial endeavours. Therefore, we hypothesise that:

- H4(a): High-quality government policies and programmes for entrepreneurs are conducive to new business creation;
- (H4b): High-quality government policies and programmes for entrepreneurs partially offset the negative effects of generous unemployment compensations on the creation of new ventures.

## Data and method

Our paper aims to investigate the relationship between the generosity of unemployment benefits schemes and the creation of new businesses in the EU countries over a period of 19 years (2001–2019). We constructed our sample based on secondary data collected from several publicly available data sources, specifically Global Entrepreneurship Monitor (GEM) database, World Bank’s World Development Indicators (WDI) database, European Commission’s Tax and Benefits Indicators (TBI) database, European Commission’s Labour Market Policies (LMP) database, and Eurostat. Because of the low availability of data, the analysis is limited to 23 EU countries^5^ for which data have been available for both the dependent and main independent variables for at least 6 years (about one-third of the total time span).

To test our hypotheses, we use two-way fixed-effects linear regression models for panel data, as depicted in Eqs. (1) and (2)

1 $$\mathrm{For}\;\mathrm{hypotheses}\;H1-H4(a):{\mathrm{Entrepr}}_{i,t}=\alpha{\mathrm{NRR}}_{i,t}+\beta_jX_{j,i,t}+\vartheta_i+\delta_t+u_{i,t}$$

2 $$\mathrm{For}\;\mathrm{hypothesis}\;H4(b):{\mathrm{Entrepr}}_{i,t}=\alpha_1{\mathrm{NRR}}_{i,t}+{\alpha_2{\mathrm{NRR}}_{i,t}\ast{\mathrm{GOV}}_{i,t}+\alpha}_3{\mathrm{GOV}}_{i,t}+\beta_jX_{j,i,t}+\vartheta_i+\delta_t+u_{i,t}$$

where:

*i*: is the country (*i* = 1–23);
*t*: is the time period (year) (*t* = 1–19);
*Entrepr*: is a measure of new business creation;
*NRR*: is a measure of unemployment benefit generosity (the net replacement rate);
$${X}_{j}$$
is a vector of control variables (other potential determinants of entrepreneurship);
*GOV*: are variables measuring the quality of government policies and programmes for entrepreneurs;
$${\vartheta }_{i}$$, $${\delta }_{t}$$, and $${u}_{i,t}$$
are the unobserved individual effects, time effects, and observation-specific errors, respectively.

Time (year) effects are introduced to capture country-invariant heterogeneity due to time. These count for events that affected all countries in a year in the same way, like the outbreak of the international economic and financial crisis of 2007–2009 or the fiscal consolidation measures adopted by the EU countries in response to high public debt levels and unsustainable budget deficits after 2010. In addition, country-fixed effects allow us to also control for unobserved heterogeneity across the EU Member States, capturing the impact of country-specific time-invariant factors like the geographical environment, cultural values, or informal institutional frameworks (Chowdhury et al., 2019). We included year dummy variables for time effects and estimated all models with the fixed effects (within) estimator. In fixed-effects models, interaction can lead to unwanted effects when both variables that interact vary within units, as in our case. Giesselmann and Schmidt-Catran (2022) demonstrate that the standard FE estimator may be biased in this instance because of unobserved effect heterogeneity, and, following their proposal, we used a “double-demeaned” interaction estimator (dd-IE) for Eq. (2). Cluster-robust standard errors were used to control for heteroskedasticity and autocorrelation, often present in panel data. Moreover, we checked for high collinearity among the explanatory variables; the highly-correlated variables, with correlation coefficients above 0.6, have not been included together in the same model (Table 1 reports the correlation coefficients for the dependent and main explanatory variables).

**Table 1:** Correlations and descriptive statistics for the dependent and main explanatory variables

	Variable	Obs	Mean	Min	Max	Std. dev	(1)	(2)	(3)	(4)	(5)	(6)	(7)	(8)	(9)	(10)	(11)
(1)	Nascent entrepreneurship	330	4.23	1.09	13.4	1.94	1.00										
(2)	Opportunity entrepreneurship	315	5.05	0.81	14.7	2.03	0.81***	1.00									
(3)	Necessity entrepreneurship	315	1.39	0.09	7.9	1.06	0.67***	0.35***	1.00								
(4)	NRR_AVG	406	48.76	12.3	81.68	16.77	− 0.38***	− 0.26***	− 0.49***	1.00							
(5)	NRR_2M	406	65.74	30.31	94.51	12.94	− 0.11*	− 0.11**	− 0.19***	0.55***	1.00						
(6)	NRR_7M	406	57.51	0	86.78	19.18	− 0.38***	− 0.26***	− 0.48***	0.80***	0.64***	1.00					
(7)	NRR_13M	406	41.31	0	81.68	26.80	− 0.39***	− 0.25***	− 0.44***	0.90***	0.28***	0.56***	1.00				
(8)	NRR_25M	406	30.48	0	81.68	24.62	− 0.25***	− 0.17***	− 0.38***	0.84***	0.17***	0.45***	0.77***	1.000			
(9)	Government support score	299	2.62	1.5	3.96	0.47	− 0.04	0.09	− 0.29***	0.47***	0.06	0.39***	0.43***	0.46***	1.00		
(10)	Government programmes score	299	2.79	1.72	3.75	0.48	0.04	0.17***	− 0.27***	0.51***	0.18***	0.45***	0.41***	0.47***	0.77***	1.0.00	
(11)	Tax policy and bureaucracy score	299	2.38	1.34	3.77	0.54	0.14**	0.34***	− 0.22***	0.30***	0.0003	0.25***	0.30***	0.28***	0.74***	0.73***	1.00

(*), (**), and (***) denote statistical significance at 10%, 5%, and 1% levels, respectively.

In entrepreneurship research, several measures of entrepreneurship have been widely used, capturing different facets of the phenomenon and stages of the entrepreneurial process, among which are self-employment, the number and density of newly created businesses, new business owners, or established business owners. In our study, we assess the dynamics of new firm creation by the nascent entrepreneurship rate (NER) from the GEM database, which measures the percentage of the working-age population (aged 18–64) who are actively involved in setting up a new business that has not yet paid salaries, wages, or other forms of payments for more than 3 months. Because it refers to one of the earliest stages of the entrepreneurial process, the start-up phase, when entrepreneurial intentions turn into action, we consider it to best capture the decision to enter entrepreneurship, which is influenced by the existing social security arrangements. In addition, to test our hypotheses about the channels through which generous unemployment benefit systems affect entrepreneurial endeavours, based on what motivates an individual to start up a new business, we use GEM data on opportunity and necessity entrepreneurship rates.^6^

In our sample, the nascent entrepreneurship rate varies substantially from one country to another and in time, between a minimum of 1.09% in Hungary in 2005 and a maximum of 13.4% in Estonia in 2017 (Table 1). Moreover, people pushed into entrepreneurship by necessity reasons are less prevalent compared to opportunity-driven ones, which is common in more developed countries; the ratio between opportunity and necessity entrepreneurs is in our sample, on average, 3.63.

While data for other entrepreneurship variables, such as self-employment, are more widely available and for a longer period, using the nascent entrepreneurship rate presents two main advantages. First, NER comes from the GEM database, which is widely recognised as a relevant source for entrepreneurship data because national-level indicators are harmonised and may be used in valid cross-country comparisons.^7^ Second, NER offers a more comprehensive assessment of the overall process of new firm creation in a country by capturing the setting up of both incorporated and unincorporated businesses. By comparison, the new businesses registered indicator from World Bank’s Entrepreneurship Survey and Database captures only the number of new limited liability corporations created in a year, and there is evidence that the effects of unemployment benefits may be different for incorporated and unincorporated businesses (Xu, 2022).

The main explanatory variable, capturing the generosity of unemployment benefit systems, is the net replacement rate (NRR), computed as the percentage ratio between the net income of an unemployed individual receiving unemployment benefits and the income he had previously earned in his paying job. The rate is determined as a simple average of the net replacement rates for all types of household compositions and all levels of previous earnings, as reported in the European Commission’s TBI database. We use both the net replacement ratios for four different unemployment spells (2, 7, 13, and 25 months: NRR_2M, NRR_7M, NRR_13M, and NRR_25M, respectively) and an overall net replacement rate computed as the average of the former (NRR_AVG), capturing the differences in both relative level and duration of unemployment compensations. In our sample, these variables display the common pattern of diminishing benefit generosity with the increase in unemployment spell; the net replacement rate decreases, on average, from 65.74% in the 2nd month of unemployment to 30.48% in the 25th month (Table 1). Although these indicators are good measures of unemployment benefit generosity, they fail to capture one important feature of unemployment benefit schemes, namely the severity of criteria that unemployed persons must fulfil to qualify for payments (Robson, 2010).

In addition, to test whether the quality of entrepreneurial framework conditions, in particular of government policies and programmes for entrepreneurs, moderates the impact of unemployment compensations on the dynamics of new business creation, three other variables from the GEM database have been considered: (1) Government support score, measuring the extent to which entrepreneurship is recognised as an important economic issue by public policies and effectively supported; (2) Tax policy and bureaucracy score, capturing the degree to which tax policies and regulations are designed to encourage new business creation and SMEs; and (3) Government programmes score, measuring the existence and quality of government programmes directly targeted to assist entrepreneurs (SMEs) at both national, regional, and local level. Therefore, these variables offer an overall assessment of the quality of three different facets of entrepreneurship policies that may contribute to enhancing or inhibiting the creation of new business ventures in a country. They range from 1 to 5, with a higher value indicating more adequate policies, supports, and programmes for entrepreneurs in a country. However, as shown in Table 1, the maximum value for each of the three scores does not exceed 4 in our sample.

The scores are computed on an annual basis from data collected through national expert surveys (NES) conducted by GEM national teams in each participating country. Every year, at least 36 carefully selected experts in each country are asked to fill out a questionnaire and answer a series of statements related to entrepreneurship framework conditions on a Likert scale, rating them from completely false to completely true. The experts are selected based on their reputation and expertise, and some must be members of government staff, policymakers, organisations that manage entrepreneurship programmes, come from Chambers of Commerce and business associations, or be in a similar professional position that would allow them to have a good knowledge and understanding of government policies and programmes for entrepreneurs.^8^

In this way, the three variables present the advantage of capturing informed (expert) judgments on the status of entrepreneurship policies and programmes in different countries. Moreover, the harmonisation of primary data collecting and processing ensures that the scores are a source of comparable data at the international level on the quality of government policies and programmes for entrepreneurs. Nevertheless, some limitations may occur because of the reduced number of experts and the inherently subjective nature of survey responses and, therefore, evaluations (for a discussion on these shortcomings, see Rietveld & Patel, 2022). In addition, the scores offer a general assessment of experts’ perception of different factors from the policy framework believed to be relevant for individuals when starting a business and, therefore, fail to acknowledge the heterogenous nature of measures, incentives, and programmes to support entrepreneurs in different countries.

In examining the empirical relationship between unemployment benefit generosity and the prevalence of nascent entrepreneurship, it is also necessary to control for other important determinants of the creation of new ventures in a country. Based on the findings in previous studies and considering data availability, five such variables (the annual GDP growth rate, the unemployment rate, foreign direct investment, the share of workforce with tertiary education, and the annual population growth rate) have been included in our baseline model, mostly capturing, among others, the characteristics of the macroeconomic and socio-demographic environment in which new entrepreneurial initiatives occur.^9^ Table A1 in the Appendix reports the description of these variables and their expected effects on the decision to start a new firm.

## Results and discussions

### Effects of unemployment benefits on new business creation

To investigate in which way the generosity of unemployment benefits affects the creation of new ventures in our selected EU countries, we estimated several regression models (their results are presented in Table 2). First, as a point of reference, only the control variables were included as regressors, with and without time effects (models 1 and 2). Second, we performed alternative regressions for several different measures of the benefit replacement rate (NRR) (models 3–7). To test our first hypothesis (H1), the overall net replacement rate (NRR_AVG) was used in model 3. This variable is meant to synthetically capture the impact of changes in both the level and duration of unemployment benefits on nascent entrepreneurship. Alternatively, in models 4–7, we used the net replacement ratios for the unemployment durations of 2, 7, 13, and 25 months (NRR_2M, NRR_7M, NRR_13M, and NRR_25M). This analysis allowed us to further investigate if changes in the level of unemployment benefits at different unemployment spells affect entrepreneurial initiative in a similar way and test our second hypothesis (H2).

**Table 2:** Unemployment benefits and nascent entrepreneurship

Dependent variable—nascent entrepreneurship	(I)	(II)	(III)	(IV)	(V)	(VI)	(VII)
***Main explanatory variable***	-	-	***NRR_AVG***	***NRR_2M***	***NRR_7M***	***NRR_13M***	***NRR_25M***
Net replacement rate of unemployment benefits (NRR)	-	-	− 0.039*** (0.012)	− 0.045** (0.019)	− 0.026*** (0.004)	− 0.0155* (0.008)	− 0.014 (0.009)
***Control variables***							
Annual GDP growth rate	0.034* (0.019)	0.028 (0.028)	0.048** (0.021)	0.035 (0.020)	0.045** (0.020)	0.049** (0.020)	0.052** (0.022)
Unemployment rate	− 0.016 (0.071)	− 0.005 (0.090)	0.046 (0.081)	0.031 (0.080)	0.040 (0.082)	0.042 (0.088)	0.040 (0.085)
Foreign direct investment	− 0.015*** (0.004)	− 0.014** (0.006)	− 0.016*** (0.005)	− 0.013** (0.006)	− 0.014** (0.006)	− 0.014** (0.006)	− 0.018*** (0.006)
Share of workforce with tertiary education	0.118*** (0.035)	0.131 (0.083)	0.177** (0.068)	0.173** (0.070)	0.168** (0.071)	0.168** (0.074)	0.190** (0.075)
Annual population growth rate	0.301 (0.281)	0.533* (0.289)	0.695** (0.246)	0.660** (0.242)	0.713*** (0.246)	0.669** (0.260)	0.759*** (0.246)
***_cons***	1.375 (1.020)	3.538** (1.674)	4.340** (1.635)	5.382** (1.958)	3.947** (1.554)	3.179* (1.679)	2.643* (2.643)
*No. of observations*	330	330	315	315	315	315	315
*Year fixed effects*	No	Yes	Yes	Yes	Yes	Yes	Yes
*Country fixed effects*	Yes	Yes	Yes	Yes	Yes	Yes	Yes
*R-squared (within)*	0.147	0.386	0.425	0.419	0.427	0.405	0.406
*Adj. R-squared (within)*	0.134	0.340	0.377	0.370	0.379	0.356	0.357

(i) columns 1 and 2 report estimation results for the baseline models, without and with time effects, respectively; columns 3–7 report estimation results for the models assessing the effects of the average net replacement rate of unemployment benefits (NRR_AVG) and the specific replacement rates at different unemployment spells (2, 7, 13, and 25 months—NRR_2M, NRR_7M, NRR_13M, and NRR_25M); (ii) the coefficients of year dummy variables have not been reported for reasons of lack of space; (iii) cluster- robust standard errors between parentheses; (iv) (*), (**), and (***) denote statistical significance at 10%, 5%, and 1% levels, respectively.

Our results point to a negative relationship between net replacement rates and nascent entrepreneurship in EU countries. The sign of NRRs’ coefficients is negative in all models, though statistically significant (at *p*-value < 0.05) only in models 3–5. This broadly supports H1: that generous unemployment benefits discourage individuals from becoming entrepreneurs. When unemployment compensations are large and offered for a long time, the opportunity costs for creating a new business are higher and there is a less entrepreneurial initiative (Parker & Robson, 2004; Koellinger & Minniti, 2009; Robson, 2010; Xu, 2022). For a given country, an increase of the average unemployment benefit replacement rate (NNR_AVG) by one percentage point results in a decrease of the nascent entrepreneurship rate with almost 0.04 percentage points, all other things being equal. This is a small but nonetheless noticeable impact, given the overall low values of nascent entrepreneurship rates in the EU Member States (average NER of 4.2% in our sample). When unemployment benefits replacement rates are included as potential determinants of new business creation, our models account for up to 43% of the total variance within countries (maximum value in model 5), which is over 4% more than in the two-way fixed effects baseline model (model 2).

When looking at the impact of unemployment compensations over different unemployment spells, we can notice that the effects are unequal in time. As unemployment durations increase, the coefficient of NRR progressively decreases and loses its statistical significance. The highest (negative) coefficient and, therefore, the most important impact on the entrepreneurial initiative is recorded for the net replacement rate of the first two months of unemployment (0.045), and this decreases to about half the value in the 7th month (0.026). Nevertheless, the explanatory power of model 5, capturing the effects of NRR_7M on new business creation, is higher than that of model 4, where NRR_2M is considered (adjusted within R-squared lower by about one percentage point in model 4 compared to model 5). Therefore, our second hypothesis is only partially supported by evidence. Although we find an unequal time pattern of effects, these seem to be stronger at shorter unemployment durations; this contrasts the conventional view of most harmful high unemployment benefits at the later stages of the unemployment spell but supports the recent-date findings of Kolsrud et al. (2018) and D’Ambrosio and Scrutinio (2022) in the unemployment literature.

The dynamics of entrepreneurial initiatives among the unemployed may provide one explanation. Practical evidence for the EU countries suggests that the likelihood of unemployed individuals to consider opening a new business is not uniform along the unemployment spell, with the share of unemployed persons who seek to work on their own being higher at short unemployment durations and then diminishing in time because of shrinking skills, professional networks, or lower personal savings (which are a particularly important source of financing for new entrepreneurs). A slight recovery may be, however, noticed for long-term unemployment spells when people realise that they might not find work and become, once again, more open to the idea of embracing an entrepreneurial career (OECD & European Union, 2019). For short unemployment durations, a larger pool of potential entrepreneurs could explain why disincentives coming from generous unemployment benefits are stronger and statistically significant. At long-term unemployment spells, when the pool of potential entrepreneurs once again raises because little economic means are available, the same disincentives may not occur. Although higher unemployment benefits could better fulfil their economic needs, unemployed individuals will be less discouraged to open a new business because of the increased need for personal fulfilment, high pressure of shrinking skills and social capital (Nichols et al., 2013), also strong fear of benefits loss and no alternative job option. Furthermore, opportunity entrepreneurs may also be more strongly influenced in their decision by short-term unemployment benefits, and they represent the majority of entrepreneurs in the EU countries where, just as in other developed countries, push motives are less prevalent. Additional insights on these dynamics and arguments are developed in Section 4.2, where we explore the specific effects on different types of entrepreneurs, depending on their motivation to start a new business.

Regarding the control variables, the results are generally in line with our expectations and the findings of other studies in the entrepreneurship literature. Their coefficients are statistically significant at the 0.05 level in most models, except for the unemployment rate. Overall, the selected control variables (time dummies included) account for almost 39% of the total variation in the nascent entrepreneurship rates within countries.

A higher GDP growth rate may strengthen the demand for goods and services and boost the creation of new businesses, which explains the positive influence of this variable on nascent entrepreneurship (as also evidenced by: Wennekers et al., 2005; Van Stel et al., 2007; Hessels et al., 2008a; Bosma & Schutjens, 2011; Røed & Skogstrøm, 2014; Thai & Turkina, 2014; Barbosa et al., 2017; Laffineur et al., 2017). According to Stel et al. (2005), this positive effect is specific to relatively richer countries, as most of the EU Member States in our sample, while the influence is usually opposite in less developed countries.

On the contrary, foreign direct investment is found to be negatively related to new firm creation, meaning that a high presence of foreign companies in a country increases competition in domestic markets and discourages entrepreneurial initiative. This crowd-out effect of foreign direct investment on business entry is clearly evidenced in the studies of Arin et al. (2015) and Albulescu and Tămăşilă (2016) (only for opportunity-driven entrepreneurship). In the case of less advanced economies, such as some Central and Eastern European countries in our sample, foreign companies may also create additional incentives to choose a paying job to the detriment of an entrepreneurial career because they pay higher wages compared to domestic firms.

In our study, we found no statistically significant linear dependence of nascent entrepreneurship on the unemployment rate. On the one hand, in times of high unemployment, more unemployed individuals consider becoming entrepreneurs for necessity reasons, and, therefore, there could be a positive relationship between unemployment rates and new business creation (Bosma & Schutjens, 2011). For the EU Member States, this is confirmed by the study of the OECD and European Union (2019), which shows that in 2008–2009 the share of unemployed who were seeking to become self-employed was above 3% of all unemployed (3.6% in 2008 and 3.3% in 2009), while the same share was well below 3% in better times (2% between 2002 and 2004 and 2.4% in 2018). On the other hand, high unemployment rates could also signal a decrease in the demand for goods and services, which reduces business opportunities and inhibits entrepreneurial activities (Parker & Robson, 2004; Arin et al., 2015; Laffineur et al., 2017). Therefore, the overall effect could be unclear, as evidenced by Wennekers et al. (2005), Koellinger and Minniti (2009), Bosma and Schutjens (2011), Arin et al. (2015), Rapp et al. (2018), and Xu (2022).

The quality and quantity of human capital matter in the equation of new firm creation. More educated individuals have more knowledge and expertise and are more likely to start a new firm; therefore, the share of workforce with tertiary education is found to be positively related to nascent entrepreneurship (in line with the results of Arenius & Minniti, 2005; Van Stel et al., 2007; Røed & Skogstrøm, 2014; Arin et al., 2015; Xu, 2022). Furthermore, looking at the supply-side determinants of entrepreneurship, a higher annual population growth rate could mean a larger pool of individuals who have the knowledge required to start a business or, on the demand side, an increased demand for goods and services and more business opportunities. Either way, as our results demonstrate, population growth positively influences new business creation (similar findings are reported by: Wennekers et al., 2005; Bosma & Schutjens, 2011; Arin et al., 2015).

### Effects of unemployment benefits on opportunity and necessity entrepreneurship

We further turn our attention to the distinction between opportunity and necessity entrepreneurship to see if generous unemployment benefits affect the creation of new businesses in different ways, depending on what motivates an individual to start up a new venture. The findings are summarised in Table 3. For both necessity and opportunity entrepreneurs, we report the effects of the average net replacement rate (NRR_AVG) and the replacement rates at two representative unemployment durations, 7 and 25 months (NRR_7M and NRR_25M, respectively).

**Table 3:** Unemployment benefits, opportunity, and necessity entrepreneurship

Dependent variable	*Opportunity entrepreneurship*			*Necessity entrepreneurship*		
	**(I)**	**(II)**	**(III)**	**(IV)**	**(V)**	**(VI)**
***Main explanatory variable***	***NRR_AVG***	***NRR_7M***	***NRR_25M***	***NRR_AVG***	***NRR_7M***	***NRR_25M***
Net replacement rate of unemployment benefits (NRR)	− 0.032** (0.015)	− 0.025*** (0.005)	− 0.009 (0.012)	− 0.003 (0.006)	− 0.002 (0.003)	− 0.002 (0.003)
***Control variables***						
Annual GDP growth rate	− 0.012 (0.043)	− 0.014 (0.043)	− 0.009 (0.044)	0.013 (0.012)	0.013 (0.012)	0.013 (0.012)
Unemployment rate	− 0.081 (0.061)	− 0.086 (0.063)	− 0.090 (0.062)	0.047 (0.047)	0.046 (0.046)	0.046 (0.046)
Foreign direct investment	− 0.014*** (0.005)	− 0.012** (0.005)	− 0.015*** (0.005)	− 0.008** (0.003)	− 0.007* (0.004)	− 0.007* (0.004)
Share of workforce with tertiary education	0.153 (0.098)	0.145 (0.100)	0.159 (0.104)	0.049** (0.023)	0.049** (0.023)	0.049** (0.023)
Annual population growth rate	0.863** (0.355)	0.872** (0.345)	0.881** (0.332)	− 0.029 (0.129)	− 0.029 (0.128)	− 0.029 (0.128)
***_cons***	4.991** (2.185)	4.920** (2.009)	3.647* (1.963)	1.304** (0.559)	1.235** (1.235)	1.235** (1.235)
*No. of observations*	300	300	300	300	300	300
*Year fixed effects*	Yes	Yes	Yes	Yes	Yes	Yes
*Country fixed effects*	Yes	Yes	Yes	Yes	Yes	Yes
*R-squared (within)*	0.354	0.364	0.343	0.236	0.236	0.236
*Adj. R-squared (within)*	0.300	0.311	0.288	0.172	0.172	0.172

(i) columns 1–3 report regression results for models with opportunity entrepreneurship as the dependent variable, while columns 4–6 for necessity entrepreneurship; in each case, the effects of the average net replacement rate of unemployment benefits (NRR_AVG) and the replacement rates at 7 and 25 months of unemployment (NRR_7M and NRR_25M) are successively considered; (ii) as GEM data are made available to the public with a 3-year delay, this analysis covers the period 2001–2018; (iii) the coefficients of year dummy variables have not been reported for reasons of lack of space; (iv) cluster-robust standard errors between parentheses; (v) (*), (**), and (***) denote statistical significance at 10%, 5%, and 1% levels, respectively.

We may first notice the negative and statistically significant coefficient of NRR_AVG in model 1, evidencing that overall higher unemployment compensations decrease the incentives to become entrepreneurs of individuals who want to exploit good business opportunities. Therefore, we find support for H3(a), which is in line with the findings of Koellinger and Minniti (2009) and Audretsch et al. (2022). Moreover, the same time pattern of diminishing effects identified in Section 4.1 is ascertained (models 2 and 3).

For opportunity entrepreneurs, more generous social security benefits may act as opportunity costs for leaving paid employment, especially since the self-employed do not always benefit from the same social security coverage as salaried workers. Alternatively, when unemployment compensations are high, opportunity entrepreneurs may self-select towards unemployment and postpone the procedures for creating a new business^10^ to benefit from additional financial capital, much needed in the early stages of the entrepreneurial process. Moreover, higher social security contributions to fund more generous social benefits (Hessels et al., 2008b) and increased costs because of higher reservation wages under generous unemployment benefit systems (Beladi & Kar, 2014) may deeply cut into expected future profits and provide additional disincentives for new entrepreneurs to enter the market.

Because of diminishing unemployment compensations and the number of beneficiaries along the unemployment spell, the level of short-term unemployment benefits is deemed to be more relevant for overall social spending and, therefore, for the amount of taxes and social contributions raised to finance this expenditure. In addition, individuals are more likely to look at short-term unemployment benefits when assessing their occupational choices and setting reservation wages. As a consequence, short-term compensations may have a stronger influence on the decision of opportunity entrepreneurs to set up a new business.

The same effects cannot be evidenced in the case of necessity entrepreneurs. The coefficients of net replacement rates in models 4–6 (Table 3) are all negative, but very small in absolute value and statistically insignificant, which seems to invalidate hypothesis H3(b).^11^ A decrease in unemployment benefit generosity in a country does not significantly increase unemployed people’s appetite for creating new businesses for necessity reasons. In other words, lower unemployment benefits fail to push individuals into entrepreneurship out of necessity, simply because this is not their first and best option. The level of income in our group of EU countries might provide an additional explanation. The necessity reason for creating new businesses is less prevalent in countries with higher income and therefore, higher individual savings and wealth, where unemployed people can leave on other means for a longer period. Moreover, most of the countries in our sample have strong social security systems, offering good conditions and additional incentives for pursuing employment (Laffineur et al., 2017) at the expense of becoming self-employed.

### The role of entrepreneurship policies and programmes

In this section we investigate the direct and moderating effects of the quality of government policies and programmes for entrepreneurs on new business creation in a country to test H4(a) and H4(b). The regression results are reported in Table 4. Models 1–3 investigate the effects of the overall quality of entrepreneurship policies and support (government support score), models 4–6 of the degree to which tax policies and regulations are designed to support entrepreneurs (tax policy and bureaucracy score), and models 7–9 more specifically capture the quality of programmes directly targeting entrepreneurs and SMEs (government programmes score).

**Table 4:** Direct and moderating effects of entrepreneurship policies

Dependent variable—*nascent entrepreneurship*	(I)	(II)	(III)	(IV)	(V)	(VI)	(VII)	(VIII)	(IX)
***Main explanatory variables***	***NRR_AVG***	***NRR_7M***	***NRR_25M***	***NRR_AVG***	***NRR_7M***	***NRR_25M***	***NRR_AVG***	***NRR_7M***	***NRR_25M***
Net replacement rate of unemployment benefits (NRR)	− 0.037** (0.014)	− 0.026*** (0.005)	− 0.015 (0.010)	− 0.036** (0.014)	− 0.027*** (0.005)	− 0.014 (0.010)	− 0.032** (0.014)	− 0.026*** (0.004)	− 0.016* (0.009)
Government support score	0.795** (0.337)	0.712** (0.333)	0.849** (0.356)	-	-	-	-	-	-
Net replacement rate of unemployment benefits * government support score	0.022 (0.059)	− 0.001 (0.047)	0.030* (0.016)	-	-	-	-	-	-
Tax policy and bureaucracy score	-	-	-	0.495* (0.282)	0.453 (0.277)	0.542* (0.273)	-	-	-
Net replacement rate of unemployment benefits * tax policy and bureaucracy score	-	-	-	0.025 (0.051)	0.008 (0.047)	0.027* (0.014)	-	-	-
Government programmes score							0.967* (0.517)	0.942* (0.519)	1.046* (0.529)
Net replacement rate of unemployment benefits * government programmes score							0.055 (0.079)	0.036 (0.042)	0.060*** (0.013)
***Control variables***									
Annual GDP growth rate	0.048 (0.028)	0.047* (0.027)	0.048 (0.029)	0.060** (0.025)	0.058** (0.024)	0.061** (0.026)	0.051* (0.026)	0.050* (0.026)	0.050* (0.026)
Unemployment rate	− 0.004 (0.075)	− 0.012 (0.072)	− 0.011 (0.080)	0.023 (0.078)	0.012 (0.075)	0.018 (0.083)	0.007 (0.069)	0.003 (0.066)	− 0.003 (0.072)
Foreign direct investment	− 0.018*** (0.006)	− 0.016** (0.008)	− 0.019*** (0.006)	− 0.019*** (0.006)	− 0.017** (0.007)	− 0.020*** (0.006)	− 0.019*** (0.006)	− 0.017** (0.007)	− 0.020*** (0.006)
Share of workforce with tertiary education	0.179*** (0.055)	0.173*** (0.059)	0.188*** (0.061)	0.193*** (0.058)	0.185*** (0.060)	0.205*** (0.064)	0.166*** (0.053)	0.162*** (0.055)	0.177*** (0.054)
Annual population growth rate	0.429* (0.223)	0.463** (0.216)	0.458* (0.229)	0.515** (0.225)	0.533** (0.223)	0.549** (0.235)	0.475** (0.223)	0.515** (0.228)	0.484** (0.229)
***_cons***	2.262 (1.768)	2.165 (1.827)	0.677 (1.718)	2.847* (1.638)	2.772* (1.567)	1.276 (1.492)	1.748 (1.879)	1.676 (1.921)	0.386 (1.832)
*No. of observations*	282	282	282	282	282	282	282	282	282
*Year fixed effects*	Yes	Yes	Yes	Yes	Yes	Yes	Yes	Yes	Yes
*Country fixed effects*	Yes	Yes	Yes	Yes	Yes	Yes	Yes	Yes	Yes
*R-squared (within)*	0.445	0.447	0.435	0.429	0.435	0.420	0.446	0.450	0.450
*Adj. R-squared (within)*	0.388	0.391	0.377	0.371	0.378	0.361	0.389	0.394	0.394

(i) columns 1–3, 4–6, and 7–9 report regression results for the direct and moderating effects of government support for entrepreneurs, the quality of tax policies and bureaucracy, and government programmes for entrepreneurs, respectively; in each case, the effects of the average net replacement rate of unemployment benefits (NRR_AVG) and the replacement rates at 7 and 25 months of unemployment (NRR_7M and NRR_25M) are successively considered; (ii) the coefficients of year dummy variables have not been reported for reasons of lack of space; (iii) cluster-robust standard errors between parentheses; (iv) (*), (**), and (***) denote statistical significance at 10%, 5%, and 1% levels, respectively.

Several important findings emerge from the data in Table 4. Generous unemployment benefits are still found to discourage new business creation. The coefficients of NRR are negative and statistically significant at the 5% level in most models; insignificant coefficients are once again reported for long-term unemployment spells (in models 3, 6, and 9, although they are statistically significant at *p* < 0.1 in the latter case). We can also notice that the coefficients of unemployment benefits for different unemployment durations display the same pattern as in Table 2; they get smaller with the increase in the unemployment spell. In addition, the coefficients of control variables generally preserve their sign and statistical significance, except for the unemployment rate, for which the impact is unclear.

The perception of high-quality entrepreneurship policies and programmes is found to be positively associated with new business creation in a country. The coefficients of the variables measuring the direct impact of entrepreneurship policy framework on nascent entrepreneurship rates are positive and statistically significant at the 10% level in most models. A statistical significance at *p* < 0.05 can be noticed in models 1–3, where the quality of entrepreneurial policies and support is assessed. Overall, we find support for hypothesis H4(a), which posits that a better institutional environment, and in particular better government policies and programmes for entrepreneurs are conducive to new business creation. The lower statistical significance may result from the high heterogeneity of policies, programmes, and measures among the EU countries and, in time, heterogeneity that our explanatory variables fail to capture (see Section 3). Different policies and programmes may target specific types of entrepreneurs, specific businesses, or stages of the business life cycle. This could explain why in other studies (Audretsch et al., 2022), similar variables are found not to have a significant effect on new firm creation. Nevertheless, tackling this heterogeneity is a close to impossible task in cross-country studies because of the limited availability of entrepreneurship data collected on a comparative basis (the GEM database is, in this respect, among the best options currently available).

Some interesting conclusions emerge when looking at the interaction terms. We find evidence of positive moderating effects of entrepreneurship policies and programmes, but only on the relationship between long-term unemployment benefits (NRR_25M) and new business creation. The effects are weak for government support score and tax policy and bureaucracy score (coefficients of moderating variables statistically significant at *p* < 0.1 in models 3 and 6) but much stronger for government programmes score (coefficient statistically significant at *p* < 0.01 in model 9). In addition, the coefficient of NRR_25M becomes statistically significant at *p* < 0.1 in model 9, which is (nonetheless, weak) evidence that in the absence of programmes directly targeted to assist entrepreneurs, there might be negative effects of more generous unemployment benefits, even at longer unemployment spells.

Therefore, we find partial support for our H4(b). High-quality policies and programmes targeting entrepreneurs that help people overcome different entrepreneurial barriers and challenges partially offset the negative effects of generous unemployment compensations on the creation of new business ventures, but these effects seem to be relevant only for long-term unemployment benefits. As people have been unemployed for a long time and the pressure of getting out of unemployment increases, determining them to reconsider an entrepreneurial career, a more supportive entrepreneurship policy framework encourages them to take action, and disincentives coming from more generous unemployment benefits become weaker. In particular, specific programmes for entrepreneurship training could help them acquire the skills and qualifications to become entrepreneurs, while other programmes could provide them with much-needed funding. This is consistent with the practice of many EU countries, where extensive programmes to support the creation of new ventures and SMEs and specific programmes targeting the transition from unemployment to self-employment are in place. In fact, the latter even seems to be prioritised on the public policy agenda (Roman et al., 2013).

## Robustness checks

In this section, we test the sensitivity of our main results with respect to alternative measures of unemployment benefit generosity and public policies for entrepreneurs, and changes in control variables. The definitions and data sources of the additional variables used for robustness checks are presented in Table A1 in the Appendix. First, we use two other measures of unemployment compensations, namely public expenditure with unemployment benefits as percentage of GDP (as in Wennekers et al., 2005; Koellinger & Minniti, 2009) and an index of unemployment support as a proxy for the average benefits of an unemployed person (as in Koellinger & Minniti, 2009). While the first macro-level variable is only a weak measure of benefit generosity and is highly correlated with the unemployment rate, the latter is presumed to capture the value of unemployment compensations at the individual level and, therefore, be a better measure of the generosity of unemployment schemes. The regression results (Table A2 in the Appendix, columns 1 and 2) show that the coefficients of the main explanatory variables preserve their signs, but they are not statistically significant. However, we should keep in mind that these variables are less adequate proxies for the generosity of unemployment compensations compared to the benefit replacement rates that we use in our study.

Second, we consider a wider measure of benefit generosity, i.e. a net replacement ratio of previous earnings that includes, unemployment benefits (unemployment insurance and assistance), housing benefits, and social assistance for the unemployed (NRL). This measure is more likely to influence overall public expenditure with unemployment protection and, therefore, taxes and contributions. Moreover, when assessing opportunity costs for choosing to become entrepreneurs, individuals may consider all benefits that are lost. At longer unemployment spells, unemployed people may not have access to unemployment insurance benefits, but social assistance could be available to them on a means-tested basis. While for shorter unemployment durations the correlation between NRL and NRR (including only unemployment benefits) is very high (a correlation coefficient of 0.87 for an unemployment duration of 7 months), this correlation is weaker for longer-term unemployment (a correlation coefficient of only 0.64 for an unemployment spell of 25 months). The results (reported in Table A2 in the Appendix, columns 3–11) reiterate our main findings regarding the negative impact of generous unemployment protection schemes on new venture creation. Moreover, the coefficients of the variables capturing the direct effect of entrepreneurial policies and programmes are positive and significant at the 5 or 10% level in all models. One interesting result is that the moderating role of overall government policies and support for entrepreneurs (government support score) and entrepreneurship programmes (government programmes score) at an unemployment duration of 25 months is stronger. This comes hand in hand with a more important and statistically significant (at *p* < 0.05) direct impact of generous benefits on new business creation for long unemployment spells. One explanation is that this variable better captures the benefits to which a person is entitled at long-term unemployment durations, as social assistance replaces unemployment insurance benefits. Moreover, social assistance might be available indefinitely, depending only on economic means.

Third, we use an alternative measure of public policies for spurring the creation of new businesses, namely public expenditure with start-up incentives, as a share of GDP. As part of government spending with active labour market policies (ALMPs), this reflects public effort towards promoting entrepreneurship among the unemployed and other disadvantaged groups through advice, training, and capital provision (subsidies) to jobseekers to open their own business. The results, reported in Table A2 in the Appendix (column 12), demonstrate, once again, the significant and negative impact of more generous unemployment benefits on new venture creation. However, we did not find a significant direct or moderating effect of public spending with start-up incentive programmes on nascent entrepreneurship. Start-up incentives are not only small in scale, but there is also mixed and inconclusive evidence in the entrepreneurship literature, especially at the macroeconomic level, on their effectiveness (Barbosa et al., 2017; Laffineur et al., 2017). Such measures are found less likely to create new employer businesses and more likely to be relevant for own-account workers (Roman et al., 2013) or to have a significant effect only on new businesses created out of necessity, as a last resort option for the unemployed (Barbosa et al., 2017; Laffineur et al., 2017). In our case, this is exactly the type of entrepreneurship less influenced by generous unemployment benefits, which could explain the nonsignificant results.

Fourth and last, we change the specification of our model by adding other regressors (other determinants of new venture creation according to the literature: availability of finance, inflation, and perception of opportunities and skills to start a new business) or removing, one by one, the control variables that we used in the core model. The regression results for the model with the average net replacement rate (NRR_AVG) as the main explanatory variable are reported in Table A3 in the Appendix, but other results are available upon request. The coefficients of NRR_AVG preserve their negative sign, value, and statistical significance at the 5% level, which demonstrates the robustness of our main result, that more generous unemployment benefits in a country discourage individuals from choosing to become entrepreneurs.

## Conclusions

Unemployment benefits are an important part of the social security systems in the European Union, and their contribution to income support and poverty prevention among the unemployed is undisputed. In addition, they may act as macroeconomic stabilisers along the business cycle, helping the economy recover during recessions. However, as theory suggests, generous unemployment benefits, which compensate for a large part of lost wages, are supplied over a long period of time and do not come with strict eligibility requirements, may have important side effects on the economy.

Our paper investigated the potential detrimental impact of unemployment compensations on new business creation (measured by the nascent entrepreneurship rate) in 23 EU countries and demonstrated the existence of such side effects. Generous benefits were found to discourage individuals from becoming entrepreneurs and inhibit new business creation, although the negative effects resulted to be only small and unequal in time. Opposite to the conventional view, our analysis indicated more detrimental effects of high unemployment compensations at short unemployment spells. Because of the larger number of beneficiaries at short unemployment durations, the level of benefits in the first months of unemployment more consistently influences the tax burden of new firms; moreover, short-term benefits are more likely to serve as a benchmark for setting reservation wages. At long-term unemployment durations, as individuals feel more need for personal fulfilment, a higher pressure of shrinking skills and diminishing social capital, and a stronger fear of benefits loss with no jobs available, they may be less discouraged by generous benefits to take action and open a new business as a last-resort option. Our findings support, with much-needed evidence on entrepreneurship, the emerging approaches in unemployment literature that call for a more balanced time profile of benefits along the unemployment spell. Nevertheless, this strand of research should be further explored and our results validated by country-level or microdata analyses.

Against common belief, we also found the negative impact of generous unemployment compensations to be higher in the case of opportunity entrepreneurs. In other words, high unemployment benefits were demonstrated to be harmful to exactly those entrepreneurs who create more jobs and contribute more to economic growth in a country. For necessity entrepreneurs, the effects were inconclusive, depending on the estimation technique. In countries with strong social security systems and a higher level of income, unemployed individuals may have more powerful incentives to pursue employment and live on other means for a long period of time; therefore, less generous unemployment benefits could fail to push them into entrepreneurship. In addition, we found evidence for a positive effect of high-quality policies and programmes targeting entrepreneurs on new business creation and a moderating effect on the relationship between unemployment benefits and nascent entrepreneurship rates at long-term unemployment spells (25 months). As the pressure of getting out of unemployment increases, making people reconsider an entrepreneurial career, a more supportive entrepreneurship policy framework and well-targeted entrepreneurship programmes encourage individuals to take action and reduce disincentives coming from generous unemployment benefits.

From a policy perspective, our results suggest that, although promoting entrepreneurship may not be the main goal of governments when designing unemployment benefit policies, public authorities should nonetheless be aware of the side effects that their choices might have. Depending on the economic and social particularities of each country and period, policymakers should choose the design features of unemployment benefit schemes that ensure an optimum balance between adequate income replacement and poverty prevention, on the one hand, and limited side effects on new venture creation and employment, on the other hand.

Even when the objective of income support prevails and generous unemployment benefits (as level and duration) seem to be the right choice, governments could limit their side effects by compensatory measures, such as imposing stricter job-search requirements or allowing unemployed individuals who choose to become entrepreneurs to keep receiving unemployment compensations, while in the process of creating their own business. This is especially important when new entrepreneurs have limited financial resources to start a new venture and have to cope with losses at the early stages of their entrepreneurial endeavours.

In addition, public authorities could seek to improve entrepreneurship framework conditions. In particular, a higher quality of entrepreneurship policies and better programmes directly targeted to assisting entrepreneurs may partially offset the detrimental effects of generous unemployment benefits at long unemployment spells on new business creation. The reform of national unemployment insurance schemes to ensure equal coverage of self-employed and wage-earning individuals should also be on the agenda of many EU countries as part of their effort to support entrepreneurship.

The main limitations of our study come from data availability. Although the GEM database is widely recognised as a reliable source of entrepreneurship data and extensively used in cross-country analyses, data are not available for all countries and years, which prevented us from expanding our panel to all EU Member States. Moreover, it does not report data on necessity and opportunity nascent entrepreneurs; therefore, we used data for total early-stage entrepreneurship as a proxy, which understates the importance of necessity entrepreneurs. Another limit comes from the general nature of our variables expressing the quality of public policies and programmes targeting entrepreneurs. These are perceptual variables, expressing experts’ assessment of different facets of the entrepreneurship policy environment. More research would be welcome on the specific policies and programs in different countries and their moderating role in the relationship between unemployment compensation and new business creation. In addition, the net replacement rates of unemployment benefits that we use in our study do not capture the eligibility criteria to qualify for payments, the minimum job search requirements, or the severity of sanctions. Nevertheless, these are important features of unemployment benefit schemes, and their effects on the transition from unemployment to entrepreneurship should be further explored.

## Supplementary Information

Below is the link to the electronic supplementary material.

Supplementary file1 (DOCX 38 KB)

## Footnotes

1 In the European Union, the European Commission is actively engaged in developing or renewing policies that target business creation by using a wide set of instruments, such as the European Social Fund (a pool of funds provided for entrepreneurs to become more flexible); the Better Entrepreneurship Policy Tool (an online tool that allows us to explore how public policy can support youth, women, migrants, and the unemployed in business creation and self-employment, as well as to support the development of social enterprises); and the European Investment Advisory Hub (an initiative that offers a single access point to a 360-degree offer of advisory and technical assistance services with the aim of strengthening Europe’s business environment). The United Nations also launched a project entitled “global initiative towards post-COVID-19 resurgence of the MSME sector” to support governments and entrepreneurs alike, as well as various institutions working to support entrepreneurship through targeted advisory and capacity-building services.

2 Unemployment insurance benefits substantially differ among EU countries in terms of eligibility criteria, amount, duration, and requirements to be fulfilled to continue receiving cash payments (see Section I in the online Appendix for an overall view). While in some countries the replacement rate between unemployment benefits and previous wage earnings is below 40%, in others it is higher than 80% and generally depends on the length of employment (contributory period). The maximum duration of payments also ranges from 2 to 3 months to over 2 years. In times of economic recession and deteriorating labour market conditions, it is general practice for governments to increase unemployment benefits as means to reduce unemployed workers’ pain and maintain their consumption (Rebollo-Sanz & Rodríguez-Planas, 2020). This was a common reaction in many EU countries during the latest economic and financial crisis. However, in the aftermath of the crisis, the decrease in unemployment benefit generosity was part of the fiscal consolidation packages aimed at reducing government debt and restoring public finance sustainability. France, Hungary, Ireland, Portugal, and Spain were just a few of the countries resorting to such consolidation measures.

3 See Section I of the online Appendix for information on self-employment coverage in EU countries.

4 Nevertheless, one should keep in mind that this distinction applies only in broad terms. In reality, there are more nuances to the relationship between the previous occupational status and opportunity and necessity entrepreneurs (for an extensive discussion, see Laffineur et al., 2017).

5 A total of 22 countries that are currently members of the EU (Austria, Belgium, Croatia, Denmark, Estonia, Finland, France, Germany, Greece, Hungary, Ireland, Italy, Latvia, Luxembourg, Netherlands, Poland, Portugal, Romania, Slovakia, Slovenia, Spain, and Sweden), and the UK, which was part of the EU over the time of our analysis.

6 GEM database does not provide data for opportunity and necessity nascent entrepreneurs, but we consider total early-stage entrepreneurial activity *(TEA)* for opportunity and necessity reasons to be good proxies. *TEA* includes nascent entrepreneurs, together with the owners of new businesses created for no longer than 42 months, and there is a high correlation (correlation coefficient of 0.92) between *NER* and *TEA* in our sample. However, one limitation could be that *TEA* understates the relative importance of emerging entrepreneurial activity out of necessity because of a lower survival rate of necessity-driven entrepreneurs (Caliendo & Kritikos, 2010).

7 Most studies in the entrepreneurship literature use the rate of self-employment as a proxy for the level of entrepreneurial activity in a country. Regarding the relationship between social security systems and entrepreneurship, this is used in the papers of Parker and Robson (2004), Kanniainen and Vesala (2005), Robson (2010), and Xu (2022). An important criticism of this approach in cross-country analyses points to the differences between countries regarding the definition and measurement of self-employment (see Robson 2010 for a more detailed presentation).

8 Detailed information on experts’ selection procedure and data collection and harmonisation process is provided in GEM methodology (https://www.gemconsortium.org/about/wiki).

9 Entrepreneurship literature emphasizes a wide variety of factors that shape the dynamics of new business creation in a country, ranging from the macroeconomic, demographic, institutional, technological, and cultural characteristics of a country, to the personal characteristics and traits of individuals aspiring to become entrepreneurs. When deciding on the specification of our regression model, more than a dozen different control variables have been considered; however, we decided not to include as controls the variables that substantially reduce the number of observations because of low data availability, have a quite high correlation coefficient with some explanatory variables of interest, or were found not to have the expected coefficient sign and not to be statistically significant.

10 We would like to thank an anonymous reviewer for suggesting this alternative explanation.

11 However, this is only part of the story. In addition to the fixed effects models, we also estimated random effects models for both nascent entrepreneurs and necessity- and opportunity-driven entrepreneurs. The results are presented in Section II of the online Appendix. We evidence that higher unemployment benefits might nevertheless discourage individuals from starting up a new business out of necessity, but this negative effect is highly dependent on the estimation technique.
