# Supplementary material for: Unemployment benefits, entrepreneurship policies, and new business creation
Source: Small Bus Econ (Dordr). 2023 Feb 28:1–26. Online ahead of print. doi: 10.1007/s11187-023-00735-9 (PMC9972338; doi:10.1007/s11187-023-00735-9)
Supplement: Supplementary file 1 — Supplementary file1 (DOCX 38 KB) [file 11187_2023_735_MOESM1_ESM.docx]

**Appendix**

Unemployment Benefits, Entrepreneurship Policies and
New Business Creation

**Author information**

**Irina Bilan***

Department of Finance, Money and Public Administration, Faculty of Economics and Business Administration, Alexandru Ioan Cuza University of Iaşi, Iaşi, Romania; e-mail: [irina.bilan@uaic.ro](mailto:irina.bilan@uaic.ro)

* Corresponding author: Blvd. Carol I no. 11, 700506 Iasi, Romania; [irina.bilan@uaic.ro](mailto:irina.bilan@uaic.ro)

**Constantin-Marius Apostoaie**

Department of Finance, Money and Public Administration, Faculty of Economics and Business Administration, Alexandru Ioan Cuza University of Iaşi, Iaşi, Romania; e-mail: [marius.apostoaie@uaic.ro](mailto:marius.apostoaie@uaic.ro)

ORCID: 0000-0002-3032-5426

**Table A1. Control variables and additional variables used for robustness checks: definitions, data sources, and expected effects**

| **Variable** | **Definition** | **Data source** | **Expected effect on new business creation^(i)^** |
| --- | --- | --- | --- |
| ***A. Control variables*** | | | |
| Annual GDP growth rate | Real GDP growth rate (annual %) | World Bank, World Development Indicators (WDI) | (+)/(-) |
| Unemployment rate | Unemployment rate, ages 15 to 74 (% of total population) | Eurostat | (+)/(-) |
| Foreign direct investment | Net inflows of foreign direct investment (% of GDP) | World Bank, World Development Indicators (WDI) | (+)/(-) |
| Share of workforce with tertiary education | Tertiary education attainment level, 15 to 64 years (%) | Eurostat | (+) |
| Annual population growth rate | Annual growth rate of population (%) | World Bank, World Development Indicators (WDI) | (+) |
| ***B. Additional variables for robustness checks*** | | | |
| Public expenditure with unemployment benefits | Public expenditure with out-of-work income maintenance and support measures (% of GDP) | European Commission, Labour Market Policy (LMP) Database | (-) |
| Unemployment support index | Determined, following the procedure of Koellinger and Minniti (2009), according to the formula: (public out-of-work income maintenance and support expenditures as % of GDP*100)/(100+unemployment rate) | Calculated by the authors based on data retrieved from European Commission, Labour Market Policy (LMP) Database, and Eurostat | (-) |
| Net replacement ratio of unemployment, housing, and social assistance benefits for the unemployed (NRL) | The ratio of unemployment, housing, and social assistance benefits received by an unemployed individual to the income earned in his previous paying job (average value for all types of household compositions and all levels of previous earnings) (%) | Calculated by the authors based on data retrieved from European Commission, Labour Market Policy (LMP) Database | (-) |
| Public expenditure with start-up incentives | Public expenditure on labour market policies (ALMPs) for start-up incentives (% of GDP) | European Commission, Labour Market Policy (LMP) Database | (+) |
| Availability of finance | Domestic credit by financial institutions to the private sector of the economy ( % of GDP) | World Bank, World Development Indicators (WDI) | (+) |
| Annual inflation rate | Inflation, consumer prices (annual %) | World Bank, World Development Indicators (WDI) | (+)/(-) |
| Perceived entrepreneurial opportunities | Share of the working-age population (18-64) who perceive good opportunities to start a new firm in the area where they live | Global Entrepreneurship Monitor (GEM) database | (+) |
| Perceived entrepreneurial skills | Share of the working-age population (18-64) who believe they have the skills and knowledge required to create a new firm | Global Entrepreneurship Monitor (GEM) database | (+) |

*Notes: (i) (+) indicates that the variable is expected to be positively associated with new business creation and* *(-) indicates that the variable is expected to be negatively associated with new business creation, based on economic theory and general findings in the entrepreneurship literature.*

**Table A2. Robustness checks – other measures of unemployment benefits generosity and public policies for entrepreneurs**

| **Dependent variable – *Nascent entrepreneurship*** | **(I)** | **(II)** | **(III)** | **(IV)** | **(V)** | **(VI)** | **(VII)** | **(VIII)** | **(IX)** | **(X)** | **(XI)** | **(XII)** |
| --- | --- | --- | --- | --- | --- | --- | --- | --- | --- | --- | --- | --- |
| ***Main explanatory variables*** | ***Public expenditure with unemployment benefits*** | ***Unemployment support index*** | ***NRL_AVG*** | ***NRL_7M*** | ***NRL_25M*** | ***NRL_AVG*** | ***NRL_7M*** | ***NRL_25M*** | ***NRL_AVG*** | ***NRL_7M*** | ***NRL_25M*** | ***NRR_AVG*** |
| Unemployment benefits | -0.484  (0.512) | -0.608  (0.563) | -0.029**  (0.011) | -0.026***  (0.005) | -0.027**  (0.012) | -0.035***  (0.011) | -0.026***  (0.005) | -0.029**  (0.014) | -0.032**  (0.012) | -0.024***  (0.006) | -0.027**  (0.013) | -0.045***  (0.015) |
| Government programmes score | - | - | 1.029*  (0.500) | 0.960*  (0.514) | 1.025**  (0.485) | - | - | - | - | - | - | - |
| Unemployment benefits*  Government programmes score | - | - | 0.062  (0.091) | 0.076  (0.063) | 0.110***  (0.037) | - | - | - | - | - | - | - |
| Government support score | - | - | - | - | - | 0.857**  (0.343) | 0.800**  (0.341) | 0.864**  (0.343) | - | - | - | - |
| Unemployment benefits*  Government support score | - | - | - | - | - | 0.063  (0.084) | 0.023  (0.062) | 0.104**  (0.046) | - | - | - | - |
| Tax policy and bureaucracy score | - | - | - | - | - | - | - | - | 0.547*  (0.282) | 0.512*  (0.278) | 0.585*  (0.299) | - |
| Unemployment benefits*  Tax policy and bureaucracy score | - | - | - | - | - | - | - | - | 0.013  (0.049) | 0.021  (0.047) | 0.023  (0.034) | - |
| Public expenditure with start-up incentives | - | - | - | - | - | - | - | - | - | - | - | 0.094  (4.402) |
| Unemployment benefits*  Public expenditure with start-up incentives | - | - | - | - | - | - | - | - | - | - | - | 0.347  (0.989) |
| ***Control variables*** |  |  |  |  |  |  |  |  |  |  |  |  |
| Annual GDP growth rate | 0.028  (0.024) | 0.027  (0.023) | 0.059**  (0.022) | 0.057**  (0.023) | 0.066***  (0.021) | 0.059**  (0.022) | 0.054**  (0.024) | 0.068*** (0.021) | 0.068***  (0.022) | 0.064***  (0.023) | 0.074***  (0.021) | 0.047*  (0.025) |
| Unemployment rate | 0.019  (0.124) | 0.016  (0.120) | 0.004  (0.073) | 0.001  (0.073) | 0.014  (0.071) | -0.008  (0.081) | -0.016  (0.081) | 0.007  (0.077) | 0.018  (0.084) | 0.010 (0.083) | 0.037  (0.082) | 0.004  (0.078) |
| Foreign direct investment | -0.013**  (0.006) | -0.013**  (0.006) | -0.018**  (0.007) | -0.017**  (0.007) | -0.018**  (0.007) | -0.017**  (0.007) | -0.017**  (0.007) | -0.017**  (0.008) | -0.018**  (0.007) | -0.018**  (0.007) | -0.018**  (0.007) | -0.020***  (0.005) |
| Share of workforce with tertiary education | 0.216**  (0.088) | 0.220**  (0.087) | 0.149**  (0.054) | 0.150***  (0.052) | 0.155***  (0.053) | 0.158**  (0.056) | 0.166***  (0.058) | 0.163**  (0.058) | 0.177***  (0.060) | 0.180*** (0.060) | 0.186*** (0.061) | 0.210**  (0.083) |
| Annual population growth rate | 0.570**  (0.235) | 0.568**  (0.236) | 0.448*  (0.230) | 0.511**  (0.237) | 0.454*  (0.224) | 0.381  (0.224) | 0.434*  (0.229) | 0.405*  (0.208) | 0.476**  (0.226) | 0.520**  (0.232) | 0.530**  (0.218) | 0.595**  (0.249) |
| ***_cons*** | 2.013 (1.650) | 2.048  (1.634) | 2.125 (2.047) | 2.108  (1.835) | 1.603  (1.887) | 2.865  (1.909) | 2.366  (1.818) | 2.074  (1.719) | 3.239*  (1.846) | 2.806*  (1.618) | 2.419  (1.777) | 4.468**  (1.795) |
| *No. of observations* | 303 | 303 | 282 | 282 | 282 | 282 | 282 | 282 | 282 | 282 | 282 | 265 |
| *R-squared (within)* | 0.384 | 0.383 | 0.440 | 0.448 | 0.445 | 0.441 | 0.442 | 0.446 | 0.420 | 0.426 | 0.417 | 0.410 |
| *Adj. R-squared (within)* | 0.330 | 0.332 | 0.383 | 0.392 | 0.388 | 0.384 | 0.385 | 0.390 | 0.361 | 0.368 | 0.358 | 0.345 |

*Notes: (i) columns 1 and 2 report the regression results when unemployment benefits generosity is captured by public expenditure with unemployment benefits and an unemployment support index; (ii) columns 3-11 consider a wider measure of benefits generosity, including housing benefits and social assistance for the unemployed (NRL) and capture both the direct effects of these benefits and moderating effects of public policies for entrepreneurs (government programmes for entrepreneurs in models 3-5, government support for entrepreneurs in models 6-8, and the quality of tax policies and bureaucracy in models 9-11); in each case, the effects of the average net replacement rate of unemployment benefits (NRL_AVG) and the replacement rates at 7 and 25 months of unemployment (NRL_7M and NRL_25M) are successively considered; (iii) column 12 reports the regression results when public spending on start-up incentive programmes is used to assess the quality of public policies for entrepreneurs; the average net replacement rate of unemployment benefits (NRR_AVG) is the dependent variable; (iv) year and country fixed effects are included in all models; (v) the coefficients of year dummy variables have not been reported for reasons of lack of space; (vi) cluster-robust standard errors between parentheses; (vii) (*), (**) and (***) denote statistical significance at 10%, 5% and 1% levels, respectively.*

**Table A3. Robustness checks – adding/removing control variables**

| **Dependent variable – *Nascent entrepreneurship*** | **(I)** | **(II)** | **(III)** | **(IV)** | **(V)** | **(VI)** | **(VII)** | **(VIII)** | **(IX)** |
| --- | --- | --- | --- | --- | --- | --- | --- | --- | --- |
| Average net replacement rate of unemployment benefits *(NRR_AVG)* | -0.039***  (0.011) | -0.038***  (0.012) | -0.037***  (0.012) | -0.039**  (0.015) | -0.040***  (0.014) | -0.036***  (0.012) | -0.037**  (0.011) | -0.034**  (0.013) | -0.034**  (0.013) |
| ***Control variables*** |  |  |  |  |  |  |  |  |  |
| Annual GDP growth rate | - | 0.038  (0.026) | 0.031**  (0.014) | 0.046*  (0.026) | 0.031  (0.022) | 0.068**  (0.029) | 0.047*  (0.023) | 0.034  (0.022) | 0.063***  (0.019) |
| Unemployment rate | 0.022  (0.081) | - | 0.040  (0.082) | 0.041  (0.076) | -0.043  (0.069) | 0.046  (0.062) | 0.032  (0.085) | 0.079  (0.082) | 0.086  (0.089) |
| Foreign direct investment | -0.014**  (0.006) | -0.016***  (0.005) | - | -0.014**  (0.006) | -0.018***  (0.004) | -0.019***  (0.005) | -0.016***  (0.005) | -0.015**  (0.005) | -0.017***  (0.006) |
| Share of workforce with tertiary education | 0.176**  (0.066) | 0.176**  (0.068) | 0.168**  (0.070) | - | 0.163**  (0.073) | 0.120**  (0.047) | 0.164***  (0.056) | 0.180** (0.067) | 0.161***  (0.055) |
| Annual population growth rate | 0.637**  (0.252) | 0.575***  (0.179) | 0.736***  (0.247) | 0.611**  (0.265) | - | 0.689***  (0.230) | 0.758**  (0.267) | 0.656**  (0.252) | 0.788**  (0.294) |
| Availability of finance | - | - | - | - | - | -0.005  (0.007) | - | - | - |
| Inflation rate | - | - | - | - | - | - | -0.116  (0.135) | - | - |
| Perceived entrepreneurial opportunities | - | - | - | - | - | - | - | 0.019*  (0.010) | - |
| Perceived entrepreneurial skills | - | - | - | - | - | - | - | - | 0.061***  (0.021) |
| ***_cons*** | 4.597**  (1.647) | 4.588*** (1.504) | 4.331**  (1.747) | 7.557***  (1.026) | 5.356***  (1.627) | 5.586***  (1.564) | 4.939  (1.375) | 3.348*  (1.830) | 1.618  (2.143) |
| *No. of observations* | 315 | 315 | 315 | 315 | 315 | 310 | 315 | 315 | 315 |
| *R-squared (within)* | 0.419 | 0.422 | 0.411 | 0.460 | 0.401 | 0.429 | 0.433 | 0.434 | 0.474 |
| *Adj. R-squared (within)* | 0.373 | 0.377 | 0.365 | 0.415 | 0.354 | 0.379 | 0.384 | 0.386 | 0.429 |

*Notes: (i) models 1-5 successively eliminate one control variable from the main regression model; models 6-9 successively add an additional control variable to the main regression model, as follows: Availability of finance (column 6), Inflation rate (column 7); Perceived entrepreneurial opportunities (column 8), and Perceived entrepreneurial skills (column 9); these additional control variables are defined in Table A1; (ii) year and country fixed effects are included in all models; (iii) the coefficients of year dummy variables have not been reported for reasons of lack of space; (iv) cluster-robust standard errors between parentheses; (v) (*), (**) and (***) denote statistical significance at 10%, 5%, and 1% levels, respectively.*
